# Supplementary material for: Almost optimal geometrically local quantum LDPC codes in any dimension
Source: Nat Commun. 2026 Feb 5;17:2389. doi: 10.1038/s41467-026-69031-w (PMC12982749; doi:10.1038/s41467-026-69031-w)
Supplement: Supplementary file 1 — Supplementary Information [file 41467_2026_69031_MOESM1_ESM.pdf]

Supplementary Material of "Almost Optimal Geometrically Local Quantum LDPC Codes in any Dimension"

CONTENTS

I. Introduction 1

II. Results 2

III. Discussion 5

IV. Methods 5

A. Preliminary and literature review 9
1. Chain Complexes 9
2. Quantum CSS Code 10
3. Square complex and its subdivision 11
4. Embedding via Subdivision 11

B. Omitted Proofs in the main text 12
1. Construction of Theorem 2 12
2. Proof of Claim 6 13
3. Verification that $\mathcal{F}$ is a chain map 14
4. Proof of Theorem 10 15
5. Proof of Theorem 11 and Theorem 12 17
a. Expansion Properties of the Generalized Repetition Code and the Generalized Surface Code 19
b. Proof of Lemma 19 for Generalized Repetition Codes 20
c. Proof of Lemma 20 for Generalized Surface Codes 20

SM A: Preliminary and literature review

In this section, we will first introduce some basic facts about chain complexes and quantum CSS codes, and their relationship. We will also formally define a square complex, and how to embed it into the Euclidean space via subdivision.

1. Chain Complexes

Chain complexes offer an intuitive structure for studying quantum CSS codes. Within this framework, we can express the properties of the CSS code using the language of chain complexes, covering aspects such as dimension, distance, and energy barrier of a given code. We mainly consider chain complexes over the finite field $\mathbb{F}_2$.

Definition 13 (Chain complex). A chain complex X consists of a sequence of vector spaces $\mathbb{F}_2^{X(i)}$ generated by sets $X(i)$, along with linear maps $\partial_i \colon \mathbb{F}_2^{X(i)} \to \mathbb{F}_2^{X(i-1)}$ known as boundary operators, where the boundary operators satisfy

$$\partial_{i-1}\partial_i = 0.$$

By considering dual maps, one can also define the dual cochain complex consisting of coboundary operators. In our context, there is a canonical basis of $\mathbb{F}_2^{X(i)}$ labeled by the elements in $X(i)$. Under this basis, the

coboundary operator $\delta_i\colon \mathbb{F}_2^{X(i)} \to \mathbb{F}_2^{X(i+1)}$ can be written as $\delta_i = \partial_{i+1}^T$. The boundary operators will satisfy:

$$\delta_{i+1}\delta_i = 0.$$

We introduce some standard definitions. Elements in the kernel of the (co)boundary operators are called (co)cycles:

$$Z_i := \ker \partial_i = \{c_i \in \mathbb{F}_2^{X(i)} : \partial_i c_i = 0\}, \quad Z^i := \ker \delta_i = \{c_i \in \mathbb{F}_2^{X(i)} : \delta_i c_i = 0\}.$$

Elements in the image of the (co)boundary operators are called (co)boundaries:

$$B_i := \operatorname{im} \partial_{i+1} = \{\partial_{i+1} c_{i+1} : c_{i+1} \in \mathbb{F}_2^{X(i+1)}\}, \quad B^i := \operatorname{im} \delta_{i-1} = \{\delta_{i-1} c_{i-1} : c_{i-1} \in \mathbb{F}_2^{X(i-1)}\}.$$

A chain is called exact if $Z_i = B_i$ for all i. We can also define an exact cochain similarly.

2. Quantum CSS Code

A quantum CSS code Q is defined by two classical codes C_x, C_z represented by their parity check matrices $H_x\colon \mathbb{F}_2^n \to \mathbb{F}_2^{m_z}$ and $H_z\colon \mathbb{F}_2^n \to \mathbb{F}_2^{m_x}$ that satisfies $H_x H_z^T = 0$. Here n, m_x, m_z corresponds to the number of qubits, X checks, and Z checks respectively. It is well known that the CSS code naturally corresponds to a chain complex as follows:

$$\mathbb{F}_2^{m_x} \xrightarrow{\delta_0 = H_z^T} \mathbb{F}_2^n \xrightarrow{\delta_1 = H_x} \mathbb{F}_2^{m_z}.$$

The X and Z logical operators correspond to the code C_x and C_z, and X and Z stabilizers correspond to the code $C_z^\perp$ and $C_x^\perp$. The code dimension is defined by $k = \dim C_x - \dim C_z^\perp = \dim C_z - \dim C_x^\perp$. The code distance $d = \min(d_x, d_z)$ where

$$d_x = \min_{c_x \in C_x - C_z^\perp} |c_x|, \quad d_z = \min_{c_z \in C_z - C_x^\perp} |c_z|.$$

We will also consider the energy barrier $\mathcal{E}$ of the system. The energy barrier is the minimum energy required to change an all-zero codeword to a nontrivial codeword by flipping one bit at a time. In our context, this energy is related to the number of violated checks. For any vector $c_x \in \mathbb{F}_2^n$, we define its X energy as $\epsilon_x(c_x) = |H_x c_x|$. A sequence of vectors $\gamma_{a\to b} = (c_0 = a, c_1, \ldots, c_t = b)$ constitutes a walk from a to b if for each $i \in [0, t]$, $|c_i - c_{i+1}| = 1$. The X energy of a walk is defined by $\epsilon_x(\gamma_{a\to b}) = \max_{c \in \gamma_{a\to b}} \epsilon_x(c)$, and the X energy barrier of $\mathcal{E}_x$ is defined by

$$\mathcal{E}_x = \min_{\gamma_{0\to c}, c \in C_x - C_z^\perp} \epsilon_x(\gamma_{0\to c}).$$

Similarly, we can define its Z energy barrier $\mathcal{E}_z$. The code's energy barrier is defined by $\mathcal{E} = \min(\mathcal{E}_x, \mathcal{E}_z)$.

We say a quantum code is a low-density parity-check (LDPC) code if each check acts with a constant number of qubits, and each qubit is acted by a constant number of checks. We call a quantum LDPC code good if it has asymptotic linear dimension and distance.

Another common way to describe a quantum CSS code Q is through its corresponding Tanner graph $\mathcal{T}(Q) = (V = V_0 \cup V_1 \cup V_2, E = E_0 \cup E_1)$, which is also a 1-simplicial complex. We will map each X check to a vertex in V_0, every qubit to a vertex in V_1, and every Z check to a vertex in V_2. E_0 consists of edges between vertices in V_0 and V_1, and there is an edge between $v_0 \in V_0$ and $v_1 \in V_1$ iff $H_z(v_0, v_1) = 1$ in the parity check matrix of C_z. We can define E_1 similarly for the parity check matrix H_x. From the Tanner graph, we will also use level 0, level 1, and level 2 vertices to refer to the X checks, qubits, and Z checks respectively.

In this paper, we also consider codes that have additional geometric structures. Specifically, these codes should have an embedding in the lattice $\mathbb{Z}^D$: each qubit and each check correspond to a specific location in

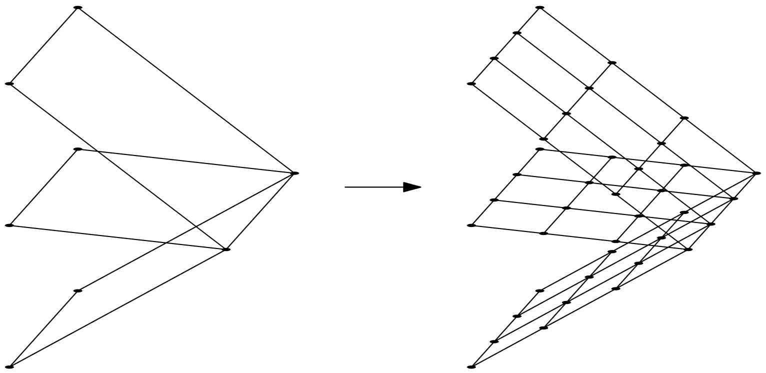

Supplementary Figure 5: L-subdivision of square complex $\mathcal{S}$ on each face f. (Figure 5 in [20])

$\mathbb{Z}^D$. We characterize the embedding map as follows:

$$I_{\text{code}\to\text{euclid}} : \text{Set}(Q) \to \mathbb{Z}^D,$$

where the $\text{Set}(Q) = [m_x] \sqcup [n] \sqcup [m_z]$ is the set of the qubits and the checks' labels. Here $[n]$ is a set of size n which labels the canonical basis vectors of $\mathbb{F}_2^n$ and $\sqcup$ is the disjoint union.

We call an embedding 'a-geometrically-local' if the Euclidean distance between each check and the qubit it interacts with is at most a in the embedding. Formally speaking, if H_{ij} is nonzero, then $|I_{\text{code}\to\text{euclid}}(i) - I_{\text{code}\to\text{euclid}}(j)| \leq a$, where $|\cdot|$ represents the Euclidean distance. Additionally, the embedding is said to have density b if the number of qubits and checks located at each lattice point in $\mathbb{Z}^D$ is at most b. Our goal is to obtain an embedding with constant parameters $a = \Theta(1)$ and $b = \Theta(1)$.

3. Square complex and its subdivision

In this section, we provide the formal definition of a square complex and its L subdivision.

Definition 14 (Square complex in 2D). A two-dimensional square complex $\mathcal{S} = (V, E, F)$ consists of a vertex set V, an edge set E, and a face set F, which satisfy the following conditions:

- For every element $e \in E$, $e = \{v_0, v_1\}$, where $v_0, v_1 \in V, v_0 \neq v_1$.
- For every element $f \in F$, $f = \{e_0, e_1, e_2, e_3\}$, where the four edges in E form a square, with four vertices in V.

We can obtain the L-subdivided complex $\mathcal{S}_L = (V_L, E_L, F_L)$ from $\mathcal{S}$ by dividing every square face $f \in F$ to an $L \times L$ grid, as shown in Supplementary Figure 5. The definition of V_L, E_L, F_L is direct from the figure.

4. Embedding via Subdivision

To embed our subdivided code into the Euclidean space $\mathbb{Z}^D$, we will use the following embedding result from [13].

Theorem 15. For any L-subdivided square complex $\mathcal{S}_L = (V_L, E_L, F_L)$ from a square complex $\mathcal{S} = (V, E, F)$, there exists an embedding map $I_{\text{square}_L\to\text{euclid}}\colon V_L \to \mathbb{Z}^D$ with constant $a, b = \Theta(1)$ such that for $L = \Theta(|V|^{\frac{1}{D-2}} \text{polylog}(|V|))$:

1. Geometrically local: For all adjacent vertices on the complex $\{v_0, v_1\} \in E_L$, the distance between corresponding points in $\mathbb{Z}^D$ is bounded, i.e. $|I_{\text{square}_L\to\text{euclid}}(v_0) - I_{\text{square}_L\to\text{euclid}}(v_1)| \leq a$.
2. Bounded density: The number of vertices at each point in $\mathbb{Z}^D$ is bounded, i.e. $\forall x \in \mathbb{Z}^D, |I^{-1}_{\text{square}_L\to\text{euclid}}(x)| \leq b$.

SM B: Omitted Proofs in the main text

1. Construction of Theorem 2

To obtain the 2D structure $\tilde{\mathcal{S}}(Q)$, we utilize the commutation relation of X and Z checks, $H_x H_z^T = 0$. The commutation relation implies that for every Z check v_0 and X check v_2 in $\mathcal{T}(Q)$, they share an even number of common neighbors $N(v_0, v_2) \subset V_1$, which allows us to pair up their common neighbors. For each pair $\{v_1, v_1'\}$, we form a square with vertices v_0, v_1, v_1', v_2. We denote the resulting face set obtained by considering all pairs of v_0, v_2 as F, and we define our resulting square complex as $\mathcal{S}(Q) = (V, E, F)$. We will refer to these faces as full faces, and they are colored in green in Supplementary Figure 2.

For certain quantum codes with parity checks H_x and H_z, we can stop here with $\mathcal{S}(Q)$ as the desired 2D structure. However, there are some pathological (or unreasonable) codes that require further treatments as we will describe them later. We say a quantum code is reasonable [18], if it doesn't have a Z/X codeword that is a subset of a Z/X stabilizer. This means as long as the distance of the code is larger than the weight, the code is reasonable. In particular, all good qLDPC codes are reasonable.

We say the stabilizer generators in H_x and H_z are 'minimal' if those stabilizers do not contain smaller stabilizers. Formally, an X-stabilizer $c_x \in C_z^\perp$ is minimal, if for all $c_x' \in C_z^\perp$ and $\operatorname{supp} c_x' \subseteq \operatorname{supp} c_x$, we have $c_x' = 0$ or c_x. Notice that if a stabilizer generator $c_x \in C_z^\perp$ from H_z is not minimal, by definition, there exist $c_x' \in C_z^\perp - 0$ and $\operatorname{supp} c_x' \subsetneq \operatorname{supp} c_x$. That means we can replace the generator c_x with c_x' and $c_x - c_x'$, while maintaining the same code C_x and C_z. Thus, given H_x and H_z, we can repeat this process until all generators are minimal. (This process terminates in finite rounds, because the stabilizer weight strictly decreases when we split the generators.) In particular, if the initial parity check matrices H_x and H_z are LDPC code, this process induces parity check matrices that are LDPC and are minimal.

The nice property of reasonable codes with minimal H_x and H_z is that the *link* of a Z/X check $v \in V_0 \cup V_2$ is always connected. This property is used later to show the subdivided code has good parameters. Let $N^F(v)$ be the faces that contain v, $N^F(v) = \{f \in F \mid v \in f\}$, and let $N^E(v)$ be the edges that contain v, $N^E(v) = \{e \in E \mid v \in e\}$. The link of v is a graph with 'vertices' $e \in N^E(v)$ and 'edges' induced by $f \in N^F(v)$ which connects the two 'vertices' $v \in e \in f$, $v \in e' \in f$. Note that for a Z check v_0, every qubit v_1 neighbor to v_0 can be identified to the 'vertex' $\{v_0, v_1\} \in N^E(v_0)$. Furthermore, every check v_2 that shares a qubit with v_0 can be identified to a set of 'edges' of the form $\{\{v_0, v_1\}, \{v_0, v_1'\}\} \in N^F(v_0)$ where v_1, v_1' are the paired qubits in the common neighbors $N(v_0, v_2)$. In particular, the endpoints of the 'edges' correspond to the shared qubits $N(v_0, v_2)$.

The following claim is highly related to [18, Lemma 6].

Claim 16. For a reasonable quantum code with minimal H_x and H_z, the link of each Z/X check $v \in V_0 \cup V_2$ is connected.

Proof. Given a Z check that corresponds to the vertex v_0, let $(V'_{link}, E'_{link}) \subseteq (V_{link}, E_{link}) = (N^E(v_0), N^F(v_0))$ be a connected component. We want to show that $(V'_{link}, E'_{link}) = (V_{link}, E_{link})$. Consider the Z operator that act on the qubits that corresponds to V'_{link}, $\{v_1 : \{v_0, v_1\} \in V'_{link}\}$. We claim that it is a logical operator. To show that, it suffices to show that it commutes with all X checks v_2 that share a qubit with v_0. As discussed above, v_2 corresponds to a set of 'edges' in $E''_{link} \subseteq E_{link}$, which acts on qubits that correspond to their endpoints. Since (V'_{link}, E'_{link}) is a connected component, the number of endpoints of E''_{link} in V'_{link} is even. Thus, the Z operator is a logical operator.

Because the code is reasonable, and the Z operator is a subset of the Z stabilizer v_0, the Z operator is a stabilizer. Because H_z is minimal, that means the operator is exactly v_0. That means $(V'_{link}, E'_{link}) = (V_{link}, E_{link})$ as desired.

For unreasonable codes or codes with non minimal H_x or H_z, however, the link of a check is not always connected. Therefore, we will include dummy faces so that the link of every check becomes connected. This is important for the subdivided code to have the desired distance property.

The corresponding 2D structure of an unreasonable code will be a relaxed notion of a square complex, which we call the square subspace complex. A square subspace complex $\tilde{\mathcal{S}} = (\tilde{V}, \tilde{E}, \tilde{F})$ also consists of vertices $\tilde{V}$, edges $\tilde{E}$, and faces $\tilde{F}$, but it is no longer required to be downward closed, i.e. a face $f \in \tilde{F}$ may contain an edge that is not in $\tilde{E}$. Generally, one can complete the square subspace complex to form a square complex, by including edges and vertices to make it downward closed. Thus, one can view a square subspace complex $\tilde{\mathcal{S}}$ as a part of a square complex $\mathcal{S}_u = (V_u, E_u, F_u)$, where $\tilde{V} \subseteq V_u$, $\tilde{E} \subseteq E_u$, and $\tilde{F} \subseteq F_u$.

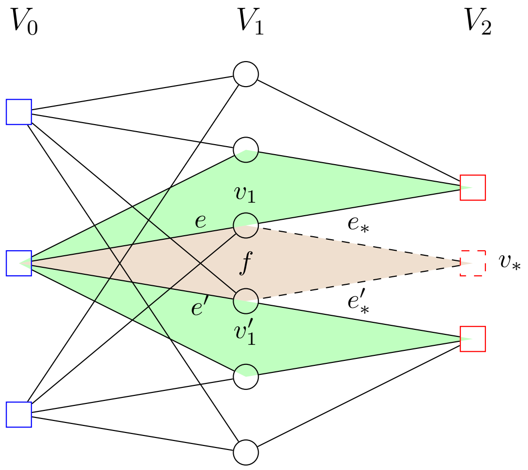

Supplementary Figure 6: A detailed version of Figure 2, where the blue, black, red vertices correspond to the X checks, qubits, Z checks respectively, we label them as V_0, V_1, V_2. We also include some of the face neighbors of one of the blue check. The green squares are the faces from $S(Q)$ which are downward closed, while the brown square is a dummy face f which contains imaginary edges e_*, e'_* and vertices v_*.

We now add dummy faces to $\mathcal{S}(Q)$ to obtain the square subspace complex $\tilde{\mathcal{S}}(Q)$ which is the desired 2D structure. The vertices and the edges are the same as before, $\tilde{V} = V$ and $\tilde{E} = E$, while there are additional faces $\tilde{F} \supseteq F$. The goal is to make the link of every check $v \in V_0 \cup V_2$ connected. To do so, we simply add a dummy face f to every pair of edges $e, e' \in N^E(v)$. The dummy face consists of two existing edges $e, e' \in \tilde{E}$ and two imaginary edges $e_* = \{v_*, v_1\}, e'_* = \{v_*, v'_1\} \notin \tilde{E}$, where $v_1, v'_1 \in V_1$, and $v_* \notin \tilde{V}$ is an imaginary vertex that is introduced to define f. An example of the dummy face f is shown in the brown part in Supplementary Figure 6. It is clear that by adding faces for every pair of edges $e, e' \in N^E(v)$, the link of v is connected.

As discussed, any good quantum LDPC code is reasonable and does not require the addition of dummy faces. However, for simplicity, we will include dummy faces regardless of whether the code is reasonable to avoid considering two separate cases. The proof in our paper relies solely on the property that the link is connected and can be easily adapted for good quantum LDPC codes without dummy faces.

2. Proof of Claim 6

This claim can be verified by counting the size of $\tilde{F}$ of our square subspace complex $\tilde{\mathcal{S}}(Q)$. Recall that $\tilde{F}$ consists of two parts, the full faces F that we obtained by pairing qubits and the dummy faces F_D we added for local edge connectivity. It is easy to see that $|F_D|$ is bounded by $\frac{\Delta^2}{2}(|X(0)| + |X(2)|)$, since for each $v \in X(0)$ or $X(2)$, we can add one dummy face between every pair of edges. To bound the size of the normal face set $|F|$, we observe that for each path $v_0 \to v_1 \to v_2$, where $v_i \in X(i)$ and is connected in the graph $\mathcal{T}(Q)$, they can determine exactly one normal face in F by our pairing algorithm. Since there are at most Δ edges from a given vertex v_0 to $X(1)$, and Δ edges for each v_1 to $X(2)$, thus for each $v_0 \in X(0)$ it can have at most Δ^2 neighboring full faces. Similar arguments also apply to vertices in $X(1)$ and $X(2)$. We can see that after the subdivision, the number of level-i vertices in X_L is increased at most $(L+1)^2/4$ times, giving our upper bound.

For general good qLDPC codes, we will have that Δ is a constant, and $|X(i)|$ are of the same order ($\frac{1}{\Delta}|X(j)| \leq |X(i)| \leq \Delta|X(j)|$ for any $i, j \in \{0, 1, 2\}$). Thus, we have $|X_L(i)| = \Theta(L^2|X(i)|)$.

Remark 17. Note that if our code is reasonable, that is we do not have the dummy face set F_D, we can directly obtain that $|X_L(i)| \leq \Delta^2 L^2 |X(i)|$. We can also observe that for each v, its face neighborhood size $|N^F(v)|$ is bounded by $\Delta^2 + \Delta^2/2 = \frac{3\Delta^2}{2}$.

3. Verification that $\mathcal{F}$ is a chain map

We will perform the verification in two steps: the first step is to verify our construction of $\mathcal{F}$ indeed satisfies the commutative diagram, thus $\mathcal{F}$ is a chain map; the second step is to verify that $Z^1(X)/B^1(X)$ is isomorphic to $Z^1(X_L)/B^1(X_L)$, which implies the code Q and code Q_L have the same dimension.

$$\begin{array}{ccccc} \mathbb{F}_2^{X(0)} & \xrightarrow{\delta_0} & \mathbb{F}_2^{X(1)} & \xrightarrow{\delta_1} & \mathbb{F}_2^{X(2)} \\ \big\downarrow \mathcal{F}_0 & & \big\downarrow \mathcal{F}_1 & & \big\downarrow \mathcal{F}_2 \\ \mathbb{F}_2^{X_L(0)} & \xrightarrow{\delta_0} & \mathbb{F}_2^{X_L(1)} & \xrightarrow{\delta_1} & \mathbb{F}_2^{X_L(2)} \end{array} \tag{B1}$$

Step One.

By our construction of $\mathcal{F}$, we can see that inside each region S, $\delta_0\mathcal{F}_0(c)|_S = 0$. Since $\mathcal{F}_1(\delta_0 c)$ will only have nonzero images in T, we can check that the commutative diagram holds inside each region S.

We proceed to check the commutation relation between different regions S, T, U. Consider the neighbor structure of the regions in X_L. Note that for the regions we specified in Supplementary Figure 3, the δ operator will be applied in the direction $S \to T \to U$.

We will first verify the commutation relation for the coboundary operators δ between U and T, i.e. $\delta_1\mathcal{F}_1 = \mathcal{F}_2\delta_1$. For each region U, consider its preimage u in $X(2)$, we can see that for each $v_1 \in X(1)$ that is connected to v, we have exactly one region T_{v_1} in X_L that is connected to U. Since the level 1 vertex that connects to U in T_v will be assigned value $c_1(v)$, this implies that $\delta_1\mathcal{F}_1 = \mathcal{F}_2\delta_1$ for the part of δ_1 implied by the edges between T and U.

Now we verify the commutation relation for coboundary operators δ implied by the edges between the regions S and T. We first note that all the level 1 vertices in S and level 2 vertices in T are assigned to 0, and each level 2 vertex in T connects to two level 1 vertices in T with the same value, thus we have $\delta_1\mathcal{F}_1 = \mathcal{F}_2\delta_1$. We can observe that the region T has the structure as shown in Supplementary Figure 7, which has a center level 1 vertex and several branches. For the center level 1 vertex of T, we observe that each δ_0 edge from a level 0 vertex connected to it will also correspond to an original δ_0 edge in X. When we are considering the branches of T, we can ignore the dummy faces, since the level 0 dummy faces are not connected to T, while level 2 dummy faces will be assigned to all zero. For each branch in T, we can observe that it corresponds to an edge from a level 1 vertex to a level 2 vertex in X. From our construction, each level 0 vertex $v_0 \in X(0)$ will have exactly one face f in S_{v_0} that is connected to this branch, and the level 0 vertices that connect to level 1 vertices in this branch are all assigned as $c_0(v_0)$. Since each level 1 vertex are assigned as $c_1(v_1) = (\delta_0 c_0)(v_1)$ we have $\delta_0\mathcal{F}_0 = \mathcal{F}_1\delta_0$.

Step Two

The goal is to show that $\mathcal{F}_1$ induces a bijection between the codewords (equivalent classes) $Z^1(X)/B^1(X)$ and $Z^1(X_L)/B^1(X_L)$ by mapping $[\widetilde{c}_1]$ to $[\mathcal{F}_1(\widetilde{c}_1)]$. To show this, we need to show

1. $\widetilde{c}_1 \in B^1(X) \implies \mathcal{F}_1(\widetilde{c}_1) \in B^1(X_L)$.
2. $\widetilde{c}_1 \in Z^1(X) \implies \mathcal{F}_1(\widetilde{c}_1) \in Z^1(X_L)$.
3. $\mathcal{F}_1(\widetilde{c}_1) \in B^1(X_L) \implies \widetilde{c}_1 \in B^1(X)$.
4. Given $c_1 \in Z^1(X_L)$, there exists $\widetilde{c}_1 \in Z^1(X)$, such that $c_1 \in [\mathcal{F}_1(\widetilde{c}_1)]$.

The first two imply that the map $[\widetilde{c}_1] \mapsto [\mathcal{F}_1(\widetilde{c}_1)]$ is well-defined and maps $Z^1(X)/B^1(X)$ to $Z^1(X_L)/B^1(X_L)$. The last two imply that the map is injective and surjective, respectively. So, overall, we get a bijection. We show the desired statements one by one.

1. $\widetilde{c}_1 \in B^1(X) \implies \mathcal{F}_1(\widetilde{c}_1) \in B^1(X_L)$. Because $\widetilde{c}_1 \in B^1(X)$ there exists $\widetilde{c}_0 \in \mathbb{F}_2^{X(0)}$ such that $\widetilde{c}_1 = \delta_1\widetilde{c}_0$. The statement then follows from the left block of the commutative diagram in Equation (B1), $\mathcal{F}_1(\widetilde{c}_1) = \mathcal{F}_1(\delta_0\widetilde{c}_0) = \delta_0\mathcal{F}_0(\widetilde{c}_0)$.

2. $\widetilde{c}_1 \in Z^1(X) \implies \mathcal{F}_1(\widetilde{c}_1) \in Z^1(X_L)$. This follows from the right block of the commutative diagram in Equation (B1), $\delta_1\mathcal{F}_1(\widetilde{c}_1) = \mathcal{F}_2(\delta_1\widetilde{c}_1) = 0$.

3. $\mathcal{F}_1(\widetilde{c}_1) \in B^1(X_L) \implies \widetilde{c}_1 \in B^1(X)$. Let $c_1 = \mathcal{F}_1(\widetilde{c}_1)$ and let $c_0 \in \mathbb{F}_2^{X_L(0)}$ be the vector that satisfies $\delta_0 c_0 = c_1$. Since $c_1|_S = 0$, c_0 takes the same value in each connected component $S_i \in \mathcal{S}$. That means $c_0 \in \operatorname{im} \mathcal{F}_0$.

Let $\widetilde{c}_0 = \mathcal{F}_0^{-1}(c_0)$. We claim that $\delta_0 c_0 = \widetilde{c}_1$. From the left block of the commutative diagram in Equation (B1), we have $\mathcal{F}_1(\delta_0\widetilde{c}_0) = \delta_0\mathcal{F}_0(\widetilde{c}_0) = \delta_0 c_0 = c_1 = \mathcal{F}_1(\widetilde{c}_1)$. Since $\mathcal{F}_1$ is injective $\delta_0 c_0 = \widetilde{c}_1$.

4. Given $c_1 \in Z^1(X_L)$*, there exists* $\widetilde{c}_1 \in Z^1(X)$*, such that* $c_1 \in [\mathcal{F}_1(\widetilde{c}_1)]$. We first construct $c_1' \in \operatorname{im}\mathcal{F}_1$ by removing the support of c_1 in S. Notice that the chain complex $X_L|_S : \mathbb{F}_2^{S\cap X_L(0)} \to \mathbb{F}_2^{S\cap X_L(1)} \to \mathbb{F}_2^{S\cap X_L(2)}$ is exact because the subcomplex $X_L|_{S_i}$ is exact for each connected component $S_i \in \mathcal{S}$. Since $\delta_1 c_1 = 0$, $c_1|_S \in Z^1(X_L|_S) = B^1(X_L|_S)$. Therefore, there exists $c_0 \in \mathbb{F}_2^{S\cap X_L(0)} = \mathbb{F}_2^{X_L(0)}$ such that $(c_1 + \delta_0 c_0)|_S = 0$. So $c_1' = c_1 + \delta_0 c_0$ is not supported in S and is only supported in T.

To show $c_1' \in \operatorname{im}\mathcal{F}_1$, we additionally need to show that c_1' takes the same value in each connected component $T_i \in \mathcal{T}$. Notice that $c_1' \in Z^1(X_L)$ which means c_1' violates no checks. Each check in T_i acts on two qubits in T_i and some other qubits in S. Since c_1' is not supported in S, the two nearby qubits in T_i take the same value. Hence, c_1' takes the same value in each connected component.

Let $\widetilde{c}_1 = \mathcal{F}_1^{-1}(c_1')$. We suffice to show $\widetilde{c}_1 \in Z^1(X)$. From the right block of the commutative diagram in Equation (B1), $\mathcal{F}_2(\delta_1\widetilde{c}_1) = \delta_1\mathcal{F}_1(\widetilde{c}_1) = \delta_1 c_1' = 0$. Since $\mathcal{F}_2$ is injective $\delta_1\widetilde{c}_1 = 0$ which concludes the proof.

4. Proof of Theorem 10

Recall that because X and X_L are symmetric, it suffices to study the coboundary expansion which we will now focus on.

a. Step 1: Construct c_1' *by removing* c_1 *in* S*, i.e. 2D cleaning.* By applying Corollary 22 to the disjoint regions of S with $\hat{f}_1 = c_1$, there exist $c_0^S = f_0$ and $c_1^S = f_1$ supported on S which satisfy

$$(a)\, c_1|_S = (\delta_0 c_0^S + c_1^S)|_S, \quad (b)\, \frac{\beta_0^{\text{sur}}}{2}|c_0^S|_S \le |c_1|_S, \quad (c)\, \beta_1^{\text{sur}}|c_1^S|_S \le |\delta_1 c_1|_S,$$
$$(d)\, \frac{\eta_0^{\text{sur}}}{2}|\delta_0 c_0^S|_T \le |c_1|_S, \quad (e)\, \eta_1^{\text{sur}}|\delta_1 c_1^S|_T \le |\delta_1 c_1|_S. \tag{B2}$$

We then set c_1' to be $c_1 + \delta_0 c_0^S$ restricted to the support on $T \cup U$. Notice that $T \cup U$ is the complement of S. So by (a),

$$c_1 + \delta_0 c_0^S = c_1^S + c_1'. \tag{B3}$$

Intuitively, c_0^S tries to clean up c_1 in S using the coboundaries $\delta_0 c_0^S$ as much as possible and c_1^S removes the remaining support in S. The rest of the vector supported in $T \cup U$ is c_1'.

The inequalities (b) and (c) bound the resource needed to remove the support of c_1 in S, which is intuitively saying that one can control the difference between c_1' and c_1. On the other hand, the inequalities (d) and (e) bound the unwanted effect created in $T \cup U$ caused by the cleaning process, which is intuitively saying that c_1' has small weight. More concretely, we have the following bounds on the weight

$$\begin{aligned} |c_1'| &= |c_1'|_T \\ &\le |\delta_0 c_0^S|_T + |c_1|_T \\ &\le \frac{2}{\eta_0^{\text{sur}}}|c_1|_S + |c_1|_T \\ &\le \frac{2}{\eta_0^{\text{sur}}}|c_1| \end{aligned} \tag{B4}$$

where the third inequality uses (d) and the last inequality uses $1 \leq \frac{2}{\eta_0^{\text{sur}}}$. Furthermore,

$$\begin{aligned}|\delta_1 c_1'| &= |\delta_1 c_1'|_T + |\delta_1 c_1'|_U \\ &\leq |\delta_1 c_1^S|_T + |\delta_1 c_1|_T + |\delta_1 c_1|_U \\ &\leq \frac{1}{\eta_1^{\text{sur}}}|\delta_1 c_1|_S + |\delta_1 c_1|_T + |\delta_1 c_1|_U \\ &\leq \frac{1}{\eta_1^{\text{sur}}}|\delta_1 c_1| \end{aligned} \tag{B5}$$

where the second inequality uses $|\delta_1 c_1'|_U = |\delta_1 c_1|_U$ because by Equation (B3), $\delta_1 c_1' - \delta_1 c_1 = \delta_1 c_1^S$ which is supported only in T. The third inequality uses (e) and the last inequality uses $1 \leq \frac{1}{\eta_1^{\text{sur}}}$.

b. Step 2: Construct c_1'' by making c_1' consistent in T, i.e. 1d cleaning. By applying Corollary 21 to the disjoint regions of T with $\hat{f}_0 = c_1'$, there exists $c_1^T = f_0$ supported on T which satisfies

$$(a)\, c_1' + c_1^T \in B^1, \quad (b)\, |c_1^T|_T \leq |c_1'|_T, \quad (c)\, \beta^{\text{rep}}|c_1^T|_T \leq |\delta_1 c_1'|_T, \quad (d)\, \eta^{\text{rep}}|\delta_1 c_1^T|_U \leq |\delta_1 c_1'|_T. \tag{B6}$$

We then set c_1'' to be $c_1' + c_1^T$ which by (a) violates no checks in T, i.e. consistent in T.

We can again bound the weight properties of c_1''

$$|c_1''| = |c_1' + c_1^T|_T \leq |c_1'|_T + |c_1^T|_T \leq 2|c_1'|_T \leq \frac{4}{\eta_0^{\text{sur}}}|c_1| \tag{B7}$$

where the third inequality uses (b) and the last inequality uses Equation (B4). Further,

$$\begin{aligned}|\delta_1 c_1''| &= |\delta_1 c_1''|_U \\ &\leq |\delta_1 c_1^T|_U + |\delta_1 c_1'|_U \\ &\leq \frac{1}{\eta^{\text{rep}}}|\delta_1 c_1'|_T + |\delta_1 c_1'|_U \\ &\leq \frac{1}{\eta^{\text{rep}}}|\delta_1 c_1'| \\ &\leq \frac{1}{\eta^{\text{rep}}\eta_1^{\text{sur}}}|\delta_1 c_1| \end{aligned} \tag{B8}$$

where the third inequality uses (d) and the last inequality uses Equation (B5).

c. Step 3: Construct $\widetilde{c}_1$ from c_1'' by moving from X_L to X. Because c_1'' is consistent on each connected region of T, we can define $\widetilde{c}_1 = \mathcal{F}_1^{-1}(c_1'') \in \mathbb{F}_2^{X(1)}$.

Because each connected region of T has size $\geq L$, we can again bound the weight properties of $\widetilde{c}_1$ where $|\widetilde{c}_1| \leq |c_1''|/L$. Furthermore, $|\delta_1 \widetilde{c}_1| = |\delta_1 c_1''|$ because all the checks in T are satisfied and by the construction of $\mathcal{F}_1$, $|\delta_1 \widetilde{c}_1|_U = |\delta_1 c_1''|_U$. (Another way to see this is to use the commutative diagram Equation (B1) $\delta_1 c_1'' = \delta_1 \mathcal{F}_1(c_1) = \mathcal{F}_2(\delta_1 \widetilde{c}_1)$. Notice that $\mathcal{F}_2$ does not change the weight of the vector by construction. Hence, $|\delta_1 \widetilde{c}_1| = |\delta_1 c_1''|$.)

d. Step 4: Construct $\widetilde{c}_0$ from $\widetilde{c}_1$ by applying expansion assumption of X. Notice the weight $\widetilde{c}_1$ is small:

$$|\widetilde{c}_1| \leq |c_1''|/L \leq \frac{4}{\eta_0^{\text{sur}} L}|c_1| \leq \frac{4}{\eta_0^{\text{sur}} L}\alpha|X_L(1)| \leq \frac{4L}{\eta_0^{\text{sur}}}\frac{\Delta_{\max}^2}{4}\alpha|X(1)| = \alpha_{\text{qLDPC}}|X(1)|$$

where the fourth inequality uses Claim 6, $|X_L(1)| \leq \Theta\left(\Delta_{\max}^2 L^2\right)|X(1)|$, and we take the constant as 1/4 as an example, as the proof will also work for other constants. Therefore, by the expansion assumption of X, there exists $\widetilde{c}_0 \in X(0)$ such that $\beta_{\text{qLDPC}}|\widetilde{c}_1 + \delta_0 \widetilde{c}_0| \leq |\delta_1 \widetilde{c}_1|$.

e. Step 5: Construct c_0'' from $\widetilde{c}_0$ by moving back from X to X_L. We lift $\widetilde{c}_0$ to $c_0'' = \mathcal{F}_0(\widetilde{c}_0) \in \mathbb{F}_2^{X_L(0)}$.

Notice that each entry of $\widetilde{c}_0$ is repeated $\leq (\Delta_{\max}\frac{L-1}{2} + 1)^2 \leq \frac{\Delta_{\max}^2}{4}L^2$ times, and each entries of $\widetilde{c}_1$ is

repeated between L and $\Delta_{\max}\frac{L-1}{2}+1 \leq \frac{\Delta_{\max}}{2}L$ times. Therefore, $\beta_{\text{qLDPC}}|\widetilde{c}_1+\delta_0\widetilde{c}_0| \leq |\delta_1\widetilde{c}_1|$ lifts into

$$\beta_{\text{qLDPC}}|c_1''+\delta_0 c_0''| \leq \frac{\Delta_{\max}}{2}L|\delta_1 c_1''| \tag{B9}$$

because $|c_1''+\delta_0 c_0''| \leq \frac{\Delta_{\max}}{2}L|\widetilde{c}_1+\delta_0\widetilde{c}_0|$ and $|\delta_1\widetilde{c}_1| = |\delta_1 c_1''|$.

f. Wrap up. We are ready to construct c_0 with the desired bound $\beta|c_1+\delta_0 c_0| \leq |\delta_1 c_1|$. This is done by utilizing the vectors we have derived from c_1 in the process above, $c_0^S, c_1^S, c_1', c_1^T, c_1'', \widetilde{c}_1, \widetilde{c}_0, c_0''$, and their associated inequalities.

We set $c_0 = c_0^S + c_0''$ and check the inequalities:

$$\begin{aligned}
|c_1+\delta_0 c_0| &= |c_1+\delta_0 c_0^S+\delta_0 c_0''| \\
&= |c_1^S+c_1'+\delta_0 c_0''| \\
&= |c_1^S+c_1^T+c_1''+\delta_0 c_0''| \\
&\leq |c_1^S|+|c_1^T|+|c_1''+\delta_0 c_0''| \\
&\leq \frac{1}{\beta_1^{\text{sur}}}|\delta_1 c_1| + \frac{1}{\beta^{\text{rep}}}|\delta_1 c_1'|_T + \frac{1}{\beta_{\text{qLDPC}}}\frac{\Delta_{\max}}{2}L|\delta_1 c_1''| \\
&\leq \frac{1}{\beta_1^{\text{sur}}}|\delta_1 c_1| + \frac{1}{\beta^{\text{rep}}\eta_1^{\text{sur}}}|\delta_1 c_1| + \frac{1}{\beta_{\text{qLDPC}}\eta^{\text{rep}}\eta_1^{\text{sur}}}\frac{\Delta_{\max}}{2}L|\delta_1 c_1| \\
&= \left(\frac{1}{\beta_1^{\text{sur}}} + \frac{1}{\beta^{\text{rep}}\eta_1^{\text{sur}}} + \frac{1}{\beta_{\text{qLDPC}}\eta^{\text{rep}}\eta_1^{\text{sur}}}\frac{\Delta_{\max}}{2}L\right)|\delta_1 c_1|
\end{aligned} \tag{B10}$$

where the second and third equality holds by construction. The fifth inequality uses Equation (B2)(c), Equation (B6)(c) and Equation (B9). The sixth inequality uses Equations (B5) and (B8). This completes the proof.

5. Proof of Theorem 11 and Theorem 12

In this subsection, we turn to prove the expansion properties of the S and T regions respectively.

We will first define the generalized repetition code and the generalized surface code according to the T and S regions, then describe their expansion properties.

The repetition code is a 1d chain of bits connected by a 1d chain of checks which require the neighboring bits to be the same. The generalized repetition code is similar, except that now there could be one branching point at the center. See Supplementary Figure 7. The adjacency matrix defines the map $\mathbb{F}_2^{Y(0)} \to \mathbb{F}_2^{Y(1)\cup Y_\partial(1)}$. This can be extended to $Y^{\text{rep}}: \mathbb{F}_2 \xrightarrow{\delta_{-1}} \mathbb{F}_2^{Y(0)} \xrightarrow{\delta_0} \mathbb{F}_2^{Y(1)\cup Y_\partial(1)}$ where δ_{-1} maps 1 to the all 1 vector. Notice that the restriction of the linear maps to the interior, $\mathbb{F}_2 \xrightarrow{\delta_{-1}|_Y} \mathbb{F}_2^{Y(0)} \xrightarrow{\delta_0|_Y} \mathbb{F}_2^{Y(1)}$, is a chain complex.

We say a generalized repetition code has length L if there are L bits on the path from one boundary to another boundary. Although the definition of length may seem arbitrary, it is defined in a way that eliminates the need for additional conversions when applying the result to the previous section.

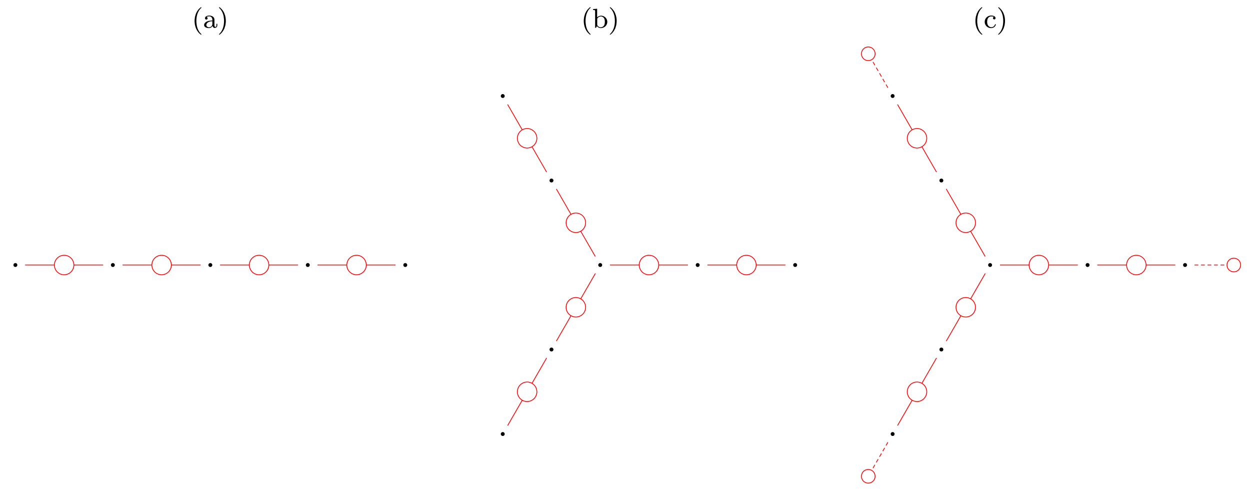

Supplementary Figure 7: The figure illustrates the generalized repetition codes of length 5. The black dots are the bits $X(0)$ and the red circles are the checks $X(1)$. (a) The standard repetition code. (b) The generalized repetition code with degree 3 branching at the center. (c) The same generalized repetition code with boundaries illustrated using the smaller circles.

For generalized surface codes, we will derive it from the local structure of S, as we will divide each face in the complex $\tilde{S}(X)$, see the example in Supplementary Figure 8. The adjacency matrices define the map $\mathbb{F}_2^{Y(0)} \to \mathbb{F}_2^{Y(1)\cup Y_\partial(1)} \to \mathbb{F}_2^{Y(2)\cup Y_\partial(2)}$ which again can be extended to $Y^{\mathrm{sur}} : \mathbb{F}_2 \xrightarrow{\delta_{-1}} \mathbb{F}_2^{Y(0)} \xrightarrow{\delta_0} \mathbb{F}_2^{Y(1)\cup Y_\partial(1)} \xrightarrow{\delta_1} \mathbb{F}_2^{Y(2)\cup Y_\partial(2)}$ where δ_{-1} maps 1 to the all 1 vector. Notice that the restriction of the linear maps on the interior, $\mathbb{F}_2 \xrightarrow{\delta_{-1}|_Y} \mathbb{F}_2^{Y(0)} \xrightarrow{\delta_0|_Y} \mathbb{F}_2^{Y(1)} \xrightarrow{\delta_1|_Y} \mathbb{F}_2^{Y(2)}$, is a chain complex. We say a generalized surface code has length L if the generalized repetition code on the seam has length L.

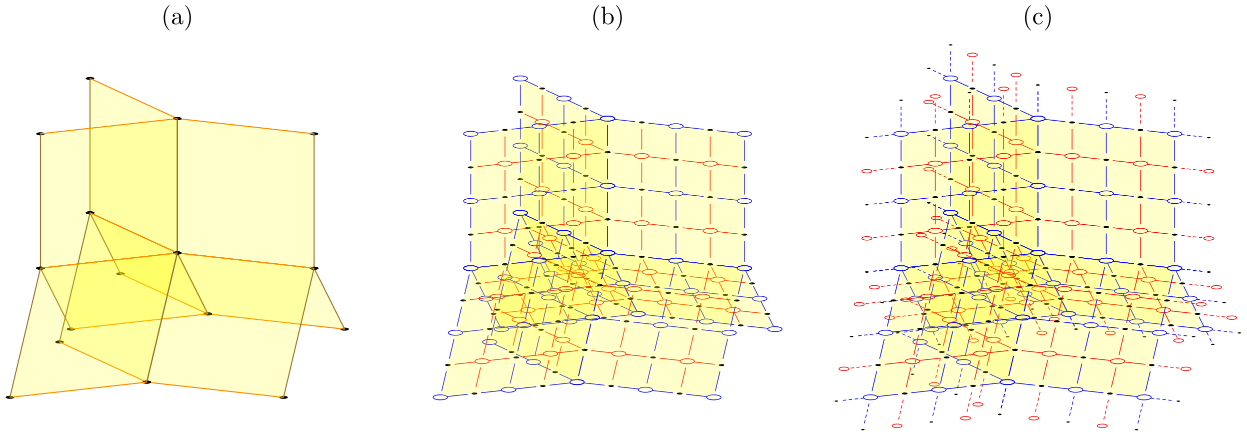

Supplementary Figure 8: The figure illustrates the generalized surface codes of length 5. The blue circles are the stabilizers $X(0)$, the black dots are the qubits $X(1)$, and the red circles are the checks $X(2)$. (a) The scaffold is the Cartesian product of two Y-shaped structure with degree 3 branching. (b) The generalized surface code under the current face structure. (c) The same generalized surface code with boundaries illustrated using the smaller circles.

a. *Expansion Properties of the Generalized Repetition Code and the Generalized Surface Code*

Having defined the generalized repetition code and generalized surface code, we now describe the desired expansion properties.

Definition 18. A chain complex Y with boundary Y_∂ is a (β_i, η_i)-coboundary expander at level i if for all $\hat{f}_i \in \mathbb{F}_2^{Y(i)}$, there exists $f_i \in \hat{f}_i + B^i \subset \mathbb{F}_2^{Y(i)}$ (recall $B^i = \operatorname{im} \delta_{i-1}|_Y$ where both the domain and the range are restricted to Y.) such that

$$(1)\, |f_i|_{\text{int}} \leq |\hat{f}_i|_{\text{int}}, \quad (2)\, \beta_i |f_i|_{\text{int}} \leq |\delta_i \hat{f}_i|_{\text{int}}, \quad (3)\, \eta_i |\delta_i f_i|_\partial \leq |\delta_i \hat{f}_i|_{\text{int}}. \tag{B11}$$

Inequality (2) is the standard coboundary expansion for Y and inequality (3) is the extra inequality which bounds the newly introduced objects during cleaning.

The following two lemmas say the generalized repetition code and the generalized surface code have coboundary expansion property. We will use the subscript to keep track of the level we are discussing.

Lemma 19. A generalized repetition code with size L, Y^{rep}, is a $(\beta_0 = \frac{2}{L}, \eta_0 = 1)$-coboundary expander at level 0.

Lemma 20. A generalized surface code with size L, Y^{sur}, is a $(\beta_0 = \Theta\left(\frac{1}{L}\right), \eta_0 = \Theta(1))$-coboundary expander at level 0 and a $(\beta_1 = \frac{2}{3L}, \eta_1 = \frac{1}{2})$-coboundary expander at level 1.

We attempt to phrase the lemmas above to elucidate the structure. However, it is not obvious how the current statements relate to the main proof presented in the previous section. So the following rephrasing seeks to enhance explicitness and usability.

Corollary 21. Given a generalized repetition code with size L, $Y^{\text{rep}} : \mathbb{F}_2 \xrightarrow{\delta_{-1}} \mathbb{F}_2^{Y(0)} \xrightarrow{\delta_0} \mathbb{F}_2^{Y(1) \cup Y_\partial(1)}$, for all $\hat{f}_0 \in \mathbb{F}_2^{Y(0)}$, there exists $f_0 \in \mathbb{F}_2^{Y(0)}$ such that

$$(a)\, \hat{f}_0 + f_0 \in B^0, \quad (b)\, |f_0|_{\text{int}} \leq |\hat{f}_0|_{\text{int}}, \quad (c)\, \beta^{\text{rep}} |f_0|_{\text{int}} \leq |\delta_0 \hat{f}_0|_{\text{int}}, \quad (d)\, \eta^{\text{rep}} |\delta_0 f_0|_\partial \leq |\delta_0 \hat{f}_0|_{\text{int}}, \tag{B12}$$

with $\beta^{\text{rep}} = \frac{2}{L}$ and $\eta^{\text{rep}} = 1$.

Proof of Corollary 21. By Lemma 19, we can find $f_0 \in \hat{f}_0 + B^0$ where f_0 satisfies the inequalities in Equation (B11) at level $i = 0$. By construction, we have (a). One easily checks that (1) $\implies$ (b), (2) $\implies$ (c), (3) $\implies$ (d).

Corollary 22. Given a generalized surface code with size L, $Y^{\text{sur}} : \mathbb{F}_2 \xrightarrow{\delta_{-1}} \mathbb{F}_2^{Y(0)} \xrightarrow{\delta_0} \mathbb{F}_2^{Y(1) \cup Y_\partial(1)} \xrightarrow{\delta_1} \mathbb{F}_2^{Y(2) \cup Y_\partial(2)}$, for all $\hat{f}_1 \in \mathbb{F}_2^{Y(1)}$, there exist $f_0 \in \mathbb{F}_2^{Y(0)}$ and $f_1 \in \mathbb{F}_2^{Y(1)}$ such that

$$(a)\, \hat{f}_1 = (\delta_0 f_0 + f_1)|_{\text{int}}, \quad (b)\, \frac{\beta_0^{\text{sur}}}{2} |f_0|_{\text{int}} \leq |\hat{f}_1|_{\text{int}}, \quad (c)\, \beta_1^{\text{sur}} |f_1|_{\text{int}} \leq |\delta_1 \hat{f}_1|_{\text{int}},$$
$$(d)\, \frac{\eta_0^{\text{sur}}}{2} |\delta_0 f_0|_\partial \leq |\hat{f}_1|_{\text{int}}, \quad (e)\, \eta_1^{\text{sur}} |\delta_1 f_1|_\partial \leq |\delta_1 \hat{f}_1|_{\text{int}}, \tag{B13}$$

with $\beta_0^{\text{sur}} = \Theta\left(\frac{1}{L}\right), \eta_0^{\text{sur}} = \Theta(1), \beta_1^{\text{sur}} = \frac{2}{3L}, \eta_0^{\text{sur}} = \frac{1}{2}$.

Proof of Corollary 22. By Lemma 20, we can write $\hat{f}_1 = f_1 + (\delta_0 \hat{f}_0)|_{\text{int}}$ for some $\hat{f}_0 \in \mathbb{F}_2^{X(0)}$ where f_1 satisfies the inequalities in Equation (B11) at level $i = 1$. And we can find $f_0 \in \hat{f}_0 + B^0$ where f_0 satisfies the inequalities in Equation (B11) at level $i = 0$. By construction, we have (a). One can check that (2) at level 1 $\implies$ (c) and (3) at level 1 $\implies$ (e). To show (b) and (d), we use (2) and (3) at level 0, $\beta_0^{\text{sur}} |f_0|_{\text{int}} \leq |\delta_0 \hat{f}_0|_{\text{int}}$ and $\eta_0^{\text{sur}} |\delta_0 f_0|_\partial \leq |\delta_0 \hat{f}_0|_{\text{int}}$ together with

$$|\delta_0 \hat{f}_0|_{\text{int}} \leq |\hat{f}_1|_{\text{int}} + |f_1|_{\text{int}} \leq 2|\hat{f}_1|_{\text{int}} \tag{B14}$$

where the last inequality uses (1) at level 1, $|f_1|_{\text{int}} \leq |\hat{f}_1|_{\text{int}}$.

The remainder of this section is devoted to showing Lemmas 19 and 20, i.e., the restated version of Theorems 11 and 12.

b. *Proof of Lemma 19 for Generalized Repetition Codes*

We observe that there are only two elements in $\hat{f}_0 + B^0$ since $B^0 = \{0, \mathbb{1}\}$ where 0 is the all 0 vector and $\mathbb{1}$ is the all 1 vector. So all is left is to figure out which choice satisfies the inequalities. We denote Δ as the degree of the branching. We call the branching point the root.

Proof of Lemma 19. We set $f_0 = \hat{f}_0$ if $|\hat{f}_0|_{\text{int}} \le |Y(0)|/2$. Otherwise, we set $f_0 = \hat{f}_0 + \mathbb{1}$. This immediately satisfies inequality (1) $|f_0|_{\text{int}} \le |\hat{f}_0|_{\text{int}}$. Notice that $|f_0|_{\text{int}} \le |Y(0)|/2$.

We now show (2) $\beta_0|f_0|_{\text{int}} \le |\delta_0\hat{f}_0|_{\text{int}}$ with $\beta_0 = \frac{2}{L}$. If $|\delta_0\hat{f}_0|_{\text{int}} \ge \Delta/2$, because $|f_0|_{\text{int}} \le |Y(0)|/2$ and $|Y(0)| = \Delta\frac{L-1}{2}+1$, the inequality is satisfied. Otherwise, $|\delta_0\hat{f}_0|_{\text{int}} < \Delta/2$, this means $\delta_0\hat{f}_0|_{\text{int}}$ is not supported on $\Delta - |\delta_0\hat{f}_0|_{\text{int}}$ branches which means these branches take the same value as the root. We observe that this value has to be 0 otherwise the root and at least half of the branches have value 1 which violates $|f_0|_{\text{int}} \le |Y(0)|/2$. Therefore, $|f_0|_{\text{int}} \le \frac{L}{2}|\delta_0\hat{f}_0|_{\text{int}}$, since f_0 can be supported on at most $|\delta_0\hat{f}_0|_{\text{int}}$ branches and each branch has weight at most $\frac{L}{2}$.

Finally, we show (3) $\eta_0|\delta_0 f_0|_\partial \le |\delta_0\hat{f}_0|_{\text{int}}$ with $\eta_0 = 1$. We divide the Δ branches into different groups based on the number of 1s of $\delta_0\hat{f}_0|_{\text{int}}$ in each branch. Let s, t, u be the number of branches with no 1, with an odd number of 1s, and an even nonzero number of 1s, respectively. Then $|\delta_0\hat{f}_0|_{\text{int}} \ge t + 2u$. If the root value of f_0 is 0, then $|\delta_0 f_0|_\partial = t$ which satisfies the inequality $|\delta_0 f_0|_\partial \le |\delta_0\hat{f}_0|_{\text{int}}$. Otherwise, the root value of f_0 is 1, then $|\delta_0 f_0|_\partial = s+u$. So it suffices to show $s \le t+u$. Notice these s branches takes the same value as the root which is 1. Since $|f_0|_{\text{int}} \le |Y(0)|/2$, this means s covers at most half of the Δ branches. Thus, $s \le t + u$.

Note that we will also consider the case when the generalized repetition code only has boundary vertices on some of its ends. It is easy to check that Lemma 19 still holds in this case.

c. *Proof of Lemma 20 for Generalized Surface Codes*

The specific structure of the generalized surface code contains generalized repetition code as a substructure. However, it is not clear to us how to use the properties of generalized repetition codes to the generalized surface codes. Therfore, we will show the level 0 and level 1 expansion in an ad hoc method through case studies. It will be appealing to have a simpler proof.

a. Level 0 Expansion Note that the level 0 expansion is essentially the isoperimetric inequalities. It is known that isoperimetric inequalities are closely related to functional inequalities. Therefore, our strategy is to use the functional inequalities of the generalized repetition codes to derive the functional inequalities for the generalized surface codes, which will then give the desired isoperimetric inequalities.

Definition 23. We say a graph with boundary $\mathcal{G} = (V, E, V^\partial)$ satisfies (C, C^∂)-functional inequalities if for all function $h : V \to \{0, 1\}$

$$\sum_{\{x,y\}\in E} |h(x) - h(y)| \ge \frac{C}{|V|} \sum_{x,y\in V} |h(x) - h(y)|, \tag{B15}$$

$$\sum_{\{x,y\}\in E} |h(x) - h(y)| \ge \frac{C^\partial}{|V^\partial|} \sum_{x\in V^\partial, y\in V} |h(x) - h(y)|. \tag{B16}$$

We first discuss how the functional inequalities and the coboundary expander are related. We then combine everything to prove the level 0 coboundary expansion of the surface code.

Let h be f_0 where f_0 is the vector with the smaller weight between $\hat{f}_0$ and $\hat{f}_0 + \mathbb{1}$. In the language of chain complexes, the two functional inequalities become

$$|\delta_0 f_0|_{\text{int}} \ge \frac{C}{|V|} 2|f_0|\Big(|V| - |f_0|\Big), \tag{B17}$$

$$|\delta_0 f_0|_{\text{int}} \ge \frac{C^\partial}{|V^\partial|}\bigg(|\delta_0 f_0|_\partial\Big(|V| - |f_0|\Big) + \Big(|V^\partial| - |\delta_0 f_0|_\partial\Big)|f_0|\bigg). \tag{B18}$$

Applying this relation, we obtain the following facts.

Claim 24. The graphs corresponding to the generalized repetition code satisfy ($C = \frac{1}{L}, C^\partial = \frac{1}{L}$)-functional inequalities.

Proof. Recall the generalized repetition code has ($\beta_0 = \frac{2}{L}, \eta_0 = 1$)-coboundary expansion. In particular, for $|f_0| \le |V|/2$

$$|\delta_0 f_0|_{\text{int}} \ge \frac{2}{L}|f_0|, \tag{B19}$$

$$|\delta_0 f_0|_{\text{int}} \ge |\delta_0 f_0|_\partial. \tag{B20}$$

Since $|V| \ge |V| - |f_0|$ and $|V^\partial| \ge |V^\partial| - |\delta_0 f_0|_\partial$, the inequalities above imply

$$|\delta_0 f_0|_{\text{int}} \ge \frac{1/L}{|V|} 2|f_0||V| \ge \frac{1/L}{|V|} 2|f_0|\Big(|V| - |f_0|\Big), \tag{B21}$$

$$\Big(\frac{L}{2} + \frac{L}{2}\Big)|\delta_0 f_0|_{\text{int}} \ge \frac{L}{2}|\delta_0 f_0|_\partial + |f_0| \tag{B22}$$

$$\ge \frac{1}{|V^\partial|}\Big(|\delta_0 f_0|_\partial |V| + |V^\partial||f_0|\Big) \tag{B23}$$

$$\ge \frac{1}{|V^\partial|}\Big(|\delta_0 f_0|_\partial\Big(|V| - |f_0|\Big) + \Big(|V^\partial| - |\delta_0 f_0|_\partial\Big)|f_0|\Big) \tag{B24}$$

where the second inequality uses $\frac{L}{2} \ge \frac{|V|}{|V^\partial|}$.

Thus, by comparing to Equations (B17) and (B18), the functional inequalities are satisfied for ($C = \frac{1}{L}, C^\partial = \frac{1}{L}$).

Claim 25. If the graph with boundary satisfies (C, C^∂)-functional inequalities, then the corresponding complex is a ($\beta_0 = C, \eta_0 = \frac{C^\partial |V|}{2|V^\partial|}$)-coboundary expander at level 0.

Proof. Let f_0 be the vector with the smaller weight between $\hat{f}_0$ and $\hat{f}_0 + \mathbb{1}$. Since $|f_0| \le |V|/2$, Equations (B17) and (B18) imply

$$|\delta_0 f_0|_{\text{int}} \ge C|f_0|, \tag{B25}$$

$$|\delta_0 f_0|_{\text{int}} \ge \frac{C^\partial |V|}{2|V^\partial|}|\delta_0 f_0|_\partial. \tag{B26}$$

We provide an example of the local structure of generalized surface codes, i.e. the region S in Supplementary Figure 9. Please note that in this paragraph, for better exposition of the functional inequalities, the vertices, edges, and faces in the figures will represent the vertices in $X(0)$, $X(1)$, $X(2)$ respectively. We will call the subgraph whose edges branch from the center of S the seam of S, as the $\mathcal{M}$ part in Supplementary Figure 9.

Note that the complex S together with its boundary S_∂ naturally induces a graph with boundary.

Lemma 26. The graph with boundary induced by S satisfies ($C = \Theta(1/L), C^\partial = \Theta(1/L)$) functional inequality.

As shown by Claim 25, if we prove the lemma beyond, we can show that S is a ($\beta_0 = \Theta(1/L), \eta_0 = \Theta(1)$)-coboundary expander at level 0. To simplify the formulae, we will use $L' = \frac{L+1}{2}$ in this section.

Proof of Lemma 26. To prove the functional inequality of S, i.e.

$$\sum_{\{x,y\}\in E} |g(x) - g(y)| \ge \frac{C}{|V|}\sum_{x,y\in V} |g(x) - g(y)|,$$

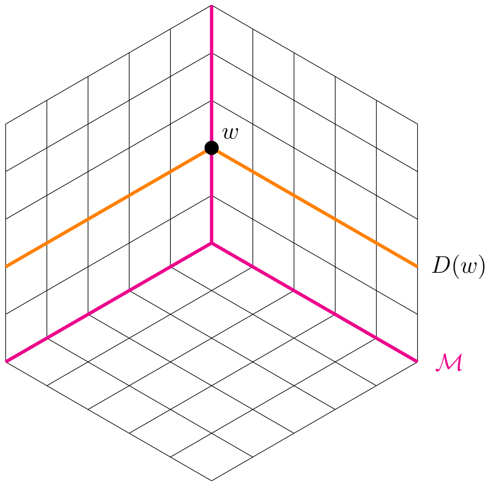

Supplementary Figure 9: An example of S with three faces (without drawing the boundary S_∂). We call the magenta part as the seam of S, denoted by $\mathcal{M}$. We also marked one generalized repetition code $D(w)$ under consideration in orange, with center w. We will use vertices, edges, and faces in the graph for level 0, 1, 2 vertices of S respectively.

we first show

$$\sum_{\{x,y\}\in E} |g(x)-g(y)| \geq \sum_{w\in\mathcal{M}} \frac{C^{rep}}{\Delta L'} \sum_{x,y\in D(w)} |g(x)-g(y)|, \tag{B27}$$

then show

$$\sum_{w\in\mathcal{M}} \sum_{x,y\in D(w)} |g(x)-g(y)| \geq \frac{1}{|N^F(v)|^2 L'} \sum_{x,y\in V} |g(x)-g(y)|, \tag{B28}$$

where we recall that $N^F(v)$ is the set of faces containing v.

Combining the two inequalities together, we obtain

$$\begin{aligned}\sum_{\{x,y\}\in E} |g(x)-g(y)| &\geq \frac{C^{rep}}{\Delta L'} \sum_{w\in\mathcal{M}} \sum_{x,y\in D(w)} |g(x)-g(y)| \\ &\geq \frac{C^{rep}}{|N^F(v)|^2 \Delta L'^2} \sum_{x,y\in V} |g(x)-g(y)|.\end{aligned}$$

Since $|N^F(v)|L'^2 \leq |V| \leq |N^F(v)|(L'+1)^2 \leq |N^F(v)|L^2$, we can observe that if we set $C = C^{rep}/2\Delta^3$,

$$\frac{C}{|V|} \leq \frac{C^{rep}}{2\Delta^3 |N^F(v)| L'^2} \leq \frac{C^{rep}}{|N^F(v)|^2 \Delta L'^2},$$

where the second inequality is by $|N^F(v)| \leq 2\Delta^2$. Therefore S satisfies functional inequality with $C = C^{rep}/2\Delta^3 = \Omega(1/L)$.

To show the first inequality (B27), we observe that the sum on the left-hand side over the edges can be decomposed to summing over the generalized repetition code $D(w)$ with center w in the seam $\mathcal{M}$, as shown in the orange part of Supplementary Figure 9. We will apply the functional inequality of generalized repetition code to each $D(w)$.

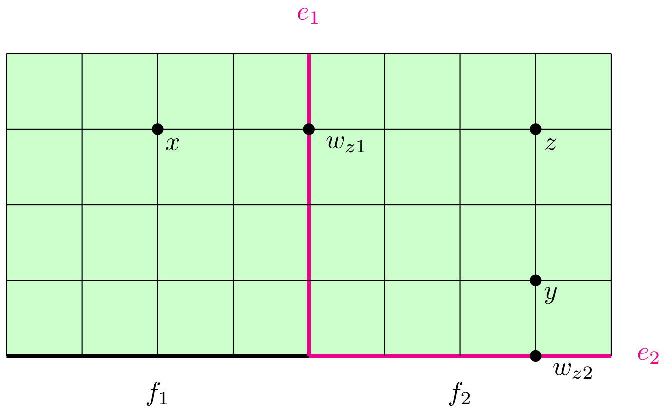

Supplementary Figure 10: The relation of x, y, z we are considering when computing C in the functional inequality. Please note that now each edge corresponds to a level 1 vertex, and each vertex corresponds to a level 0 vertex. The thickened edges are the seam of local structure S. We marked the e_1 and e_2 in our formula with red, and the faces f_1,f_2 with green.

From the observation above, we can obtain the following inequality

$$\begin{aligned}\sum_{\{x,y\}\in E} |g(x)-g(y)| &= \sum_{w\in\mathcal{M}} \sum_{\{x,y\}\in E(D(w))} |g(x)-g(y)| \\ &\geq \sum_{w\in\mathcal{M}} \frac{C^{rep}}{\Delta L'} \sum_{x,y\in D(w)} |g(x)-g(y)|,\end{aligned}$$

where the inequality is by the functional inequality of the generalized repetition code.

To show the second inequality (B28)

$$\sum_{w\in\mathcal{M}} \sum_{x,y\in D(w)} |g(x)-g(y)| \geq \frac{1}{|N^F(v)|^2 L'} \sum_{x,y} |g(x)-g(y)|,$$

we can first prove that for two neighboring faces f_1, f_2,

$$\begin{aligned}\sum_{x\in f_1, y\in f_2} |g(x)-g(y)| &\leq \sum_{z\in f_2} L' \left(\sum_{x\in f_1\cap D(w_{z1})} |g(x)-g(z)| + \sum_{y\in f_2\cap D(w_{z2})} |f(z)-f(y)| \right) \\ &\leq L' \left(\sum_{w\in e_1} \sum_{x\in D(w)\cap f_1, z\in D(w)\cap f_2} |g(x)-g(z)| + \sum_{w\in e_2} \sum_{y,z\in D(w)\cap f_2} |g(y)-g(z)| \right),\end{aligned} \tag{B29}$$

where z and x are on the same horizontal line, and z and y are on the same vertical line. Readers can refer to Supplementary Figure 10 for the definition of the notations used in the inequality. The first line is based on the triangular inequality, and the second line is by reorganizing the sum order.

For each pair of faces, we can obtain the same bound. If we sum all possible face pairs together, we will sum over all possible e_1, e_2 in the seam $\mathcal{M}$ on the right-hand side of the inequality (B29), giving us the following inequality:

$$\sum_{f,f'\in N^F(v)} \sum_{x\in f, y\in f'} |g(x)-g(y)| \leq |N^F(v)|^2 L' \sum_{w\in\mathcal{M}} \sum_{x,y\in D(w)} |g(x)-g(y)|.$$

Since the summation on the left-hand side is same as the summation over $x, y \in V$, this implies inequality (B28).

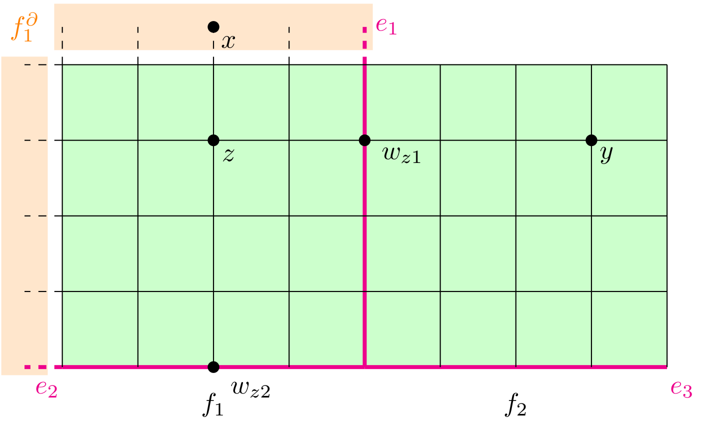

Supplementary Figure 11: The relation of x, y, z we are considering when computing C^{∂}. Note that here w_{z1} will correspond to the first term in the inequality (B30), and w_{z2} will correspond to the second term. The case when $x \in f_1^{\partial}, y \in f_1$ is similar.

For the bound on C^{∂}, we can first assume that every face f has the boundary vertices. Since every local complex S has at least one face with boundary, by adding boundary vertices to dummy faces, the $|V^{\partial}|$ will increase by at most a constant factor, and the sum $\sum_{x\in V^{\partial}, y\in V} |g(x) - g(y)|$ will only increase. Thus if we prove a lower bound for the full boundary case, we can get general lower bounds on C^{∂} by losing a constant factor.

The proof has a similar flavor as the proof above. For the boundary vertices $x \in f_1^{\partial}$ and vertices $y \in f_1$, by triangular inequality, we have

$$\sum_{x\in f_1^{\partial}, y\in f_1} |g(x) - g(y)| \le \sum_{z\in f_1} \left(L'|g(x) - g(z)| + \sum_{y\in f_1} |g(y) - g(z)| \right), \tag{B30}$$

where z and x are on the same horizontal/vertical line, and z and y are on the same vertical/horizontal line.

For $x \in f_1^{\partial}$ and $y \in f_2$ on neighboring faces f_1 and f_2, combining the inequality above, we have that

$$\begin{aligned}\sum_{x\in f_1^{\partial}, y\in f_2} |g(x) - g(y)| &\le \frac{1}{L'^2}\left(L'^2 \sum_{x\in f_1^{\partial}, z\in f_1} |g(x) - g(z)| + 2L' \sum_{z\in f_1, y\in f_2} |g(z) - g(y)| \right) \\ &= \sum_{x\in f_1^{\partial}, z\in f_1} |g(x) - g(z)| + \frac{2}{L'} \sum_{z\in f_1, y\in f_2} |g(z) - g(y)|,\end{aligned}$$

where the first inequality is by the triangular inequality over all $z \in f_1$. For the first summation term on the right-hand side, we apply inequality (B30), and for the second summation term, we apply inequality (B29). Combining the two inequalities, we have that

$$\begin{aligned}\sum_{x\in f_1^{\partial}, y\in f_2} |g(x) - g(y)| \le L' \sum_{w\in E(f_1)} \sum_{\substack{x\in D(w)\cap f_1^{\partial} \\ z\in D(w)\cap f_1}} |g(x) - g(z)| + 2 \sum_{w\in e_1} \sum_{z,y\in D(w)} |g(z) - g(y)| \\ + 2 \sum_{w\in e_3} \sum_{y,z\in D(w)\cap f_2} |g(y) - g(z)|,\end{aligned}$$

where we used $E(f_1)$ to denote the seam boundary of face f_1. For example, the branches e_1 and e_2 form the seam boundary of f_1 in Supplementary Figure 11. Readers can refer to the figure for the relation between x, y, z. The first inequality comes from triangular inequality, the second inequality is based on the previous inequality (B29) on f_1 and f_2.

Summing all (f_i^∂, f_j) pairs, we have that

$$\sum_{x\in V^\partial, y\in V} |g(x)-g(y)| \leq |N^F(v)|^2 L' \sum_{w\in\mathcal{M}} \sum_{x\in D(w)^\partial y\in D(w)} |g(x)-g(y)| + 3|N^F(v)|^2 \sum_{w\in\mathcal{M}} \sum_{x,y\in D(w)} |g(x)-g(y)|$$
$$\leq \frac{|N^F(v)|^2 L'\Delta}{C^{rep,\partial}} \sum_{\{x,y\}\in E} |g(x)-g(y)| + \frac{3|N^F(v)|^2 L'\Delta}{C^{rep}} \sum_{\{x,y\}\in E} |g(x)-g(y)|,$$

Since $|V^\partial| = 3|N^F(v)|L' \leq 6\Delta^2 L'$, we have that $C^\partial = \Omega(1/L)$.

Remark 27. Note that the result also holds for the subdivision of reasonable codes without dummy faces. The proof utilizes the fact that the link of a check is connected, which is automatically satisfied by the reasonable code.

b. Level 1 *Expansion* As discussed, the level 1 expansion is proven in an ad hoc manner.

Proof of Lemma 20 at level 1. The idea is to decompose the support of $\hat{f}_1$ into components. (For now, one can think of these as the connected components.) Because of the triangle inequality, it is sufficient to establish the desired bound, (1), (2), and (3) in Equation (B11), for each component individually. It is straightforward to show the desired bound for the component that is supported on a flat region. The remaining challenge is to show the desired bound for the components that crosses the seam which will be shown at the end. We refer to the lines at intersection of the planes as the seam.

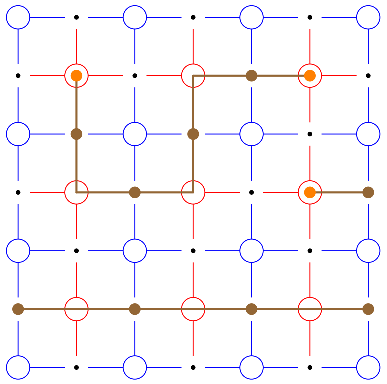

Supplementary Figure 12: The figure illustrate an example of $\hat{f}_1$. The brown dots are the support of $\hat{f}_1$. The orange dots are the violated checks, i.e. the support of $\delta_1 \hat{f}_1|_{\text{int}}$. The graph consist of edges connecting the support of $\hat{f}_1$ with the nearby checks. We see that the graph decomposes into disjoint $\hat{f}_{1,i}$.

c. Step 1: Decompose $\hat{f}_1$ into components. The goal of this step is to write $\hat{f}_1 = \sum_i \hat{f}_{1,i}$ where supp$\hat{f}_1$ is a disjoint union of supp$\hat{f}_{1,i}$. Additionally, the support of $\hat{f}_{1,i}$ will look like 1d paths which will be made precise later.

To obtain the decomposition, we consider the (bipartite) graph consisting of edges formed by the active qubits in supp$\hat{f}_1$ and their nearby checks. We call a check an active check if it is next to an active qubit. We see that a non-violated check has an even degree while a violated check has an odd degree. See Supplementary Figure 12. Based on this structure of the graph, we can decompose the graph into components whose end points are the violated checks or boundary qubits and whose active check has degree at most two. Note that when a check has more than two neighboring active qubits, there is more than one possible decomposition satisfying the above properties. In such cases, any such decomposition works. Notice that when the component does not contain qubits on the seam, the component is either a 1d loop or a 1d path which are simple objects to analyze directly.

To be more mathematical, we can write $\hat{f}_1 = \sum_i \hat{f}_{1,i}$ where $\{\hat{f}_{1,i}\}_i$ (the active qubits) have disjoint supports and $\{\delta_1 \hat{f}_{1,i} \mid_{\text{int}}\}_i$ (the violated checks) have disjoint supports. Furthermore, for each $\hat{f}_{1,i}$:

- The graph formed by the qubits and their neighboring checks, $\{(v,w) \in X(1) \times X(2) : v \in \mathrm{supp}\hat{f}_{1,i}, v \text{ is adjacent to } w\}$, is connected.

- Each check w in the graph has degree at most two.

An example of the decomposition is illustrated in Supplementary Figure 12(a).

Note that to find f_1, it suffices to find $f_{1,i}$ for each $\hat{f}_{1,i}$. This is because once given $f_{1,i}$, we can set $f_1 = \sum_i f_{1,i}$. By construction, we have $f_1 \in \hat{f}_1 + B^1$ because $f_{1,i} \in \hat{f}_{1,i} + B^1$. The inequalities also carry over to f_i. For example, to show inequality (1) in Equation (B11), we have

$$|f_1| \leq \sum_i |f_{1,i}| \leq \sum_i |\hat{f}_{1,i}| = |\hat{f}_1| \tag{B31}$$

where the first inequality holds by the triangle inequality, the second inequality, $|f_{1,i}| \leq |\hat{f}_{1,i}|$, is based on the reduced problem for $\hat{f}_{1,i}$, and the third inequality holds because $\{\hat{f}_{1,i}\}_i$ have disjoint supports. Inequalities (2) and (3) in Equation (B11) can be obtained similarly using the fact that $\{\delta_1 \hat{f}_{1,i}\mid_{\mathrm{int}}\}_i$ have disjoint supports.

d. Step 2: Bound each component. We now find $f_{1,i}$ for four different cases of $\hat{f}_{1,i}$. The last case is the hardest.

Case 1: The cluster has no violated check, i.e. $\delta_1 \hat{f}_{1,i} = 0$. In this case, one can simply set $f_{1,i} = 0$ which satisfies (1), (2), and (3).

Case 2: The cluster is not connected to the boundary nor the seam (excluding Case 1). The corresponding graph of $\hat{f}_{1,i}$ is a path that connects two violated checks. We can set $f_{1,i}$ to be a shortest path between the two violated checks, for example, the path that goes vertically then horizontally.

We now check (1), (2), and (3). Inequality (1) is satisfied because $f_{1,i}$ is the vector with the smallest weight in $\hat{f}_{1,i} + B^1$. Inequality (2) holds because $|f_{1,i}| \leq L$ and $|\delta_1 \hat{f}_{1,i}|_{\mathrm{int}} = 2$. Inequality (3) holds because $|\delta_1 f_{1,i}|_\partial = 0$. (In particular, the inequalities hold whenever $\beta_1 \leq \frac{2}{L}$.)

Case 3: The cluster is connected to the boundary but not the seam (excluding Case 1). The corresponding graph of $\hat{f}_{1,i}$ is a path that connects one violated check and the boundary. We can set $f_{1,i}$ to be a shortest path between the violated check and the boundary which either goes up vertically or right horizontally.

We now check (1), (2), and (3). Inequality (1) is satisfied because $f_{1,i}$ is the vector with the smallest weight in $\hat{f}_{1,i} + B^1$. Inequality (2) holds because $|f_{1,i}| \leq L/2$ and $|\delta_1 \hat{f}_{1,i}|_{\mathrm{int}} = 1$. Inequality (3) holds because $|\delta_1' f_{1,i}|_\partial = |\delta_1 \hat{f}_{1,i}|_{\mathrm{int}} = 1$ (In particular, the inequalities hold whenever $\beta_1 \leq \frac{2}{L}, \eta_1 \leq 1$.)

Case 4: Otherwise, the cluster is connected to the seam. The corresponding graph of $\hat{f}_{1,i}$ is more complicated, so we cannot apply structure results directly as above. What we do here is to reduce $\hat{f}_{1,i}$ to another vector $\hat{f}'_{1,i}$ that is supported next to the seam. (The region next to the seam is depicted in Supplementary Figure 13.) This is done by cleaning up the surface region using ideas similar to those in Case 1, 2, 3, and pushing the support next to but not past the seam. Vector $\hat{f}'_{1,i}$ will then be analyzed in the next step.

It is straightforward to check that the structure of the graph in the 2D area is either:

- A path that connects two qubits on the seam.

- A path that connects one qubit on the seam and a violated check.

- A path that connects one qubit on the seam and the boundary.

This allows us to decompose $\hat{f}_{1,i}$ into $\hat{f}_{1,i} = \hat{f}^x_{1,i} + \sum_j \hat{f}^y_{1,i,j} + \sum_k \hat{f}^z_{1,i,k} + \sum_l \hat{f}^w_{1,i,l}$ with disjoint support where

- $\hat{f}^x_{1,i}$ is supported on the seam.

- $\hat{f}^y_{1,i,j}$ are the parts that connect two qubits on the seam.

- $\hat{f}^z_{1,i,k}$ are the parts that connect one qubit on the seam to the boundary.

- $\hat{f}^w_{1,i,l}$ are the parts that connect one qubit on the seam to a violated check.

Furthermore, $\{\ |\ \delta_1 f^z_{1,i,k}|_\partial\ \}_k$ are disjoint and $\{\ |\ \delta_1 f^w_{1,i,l}|_{\text{int}}\ \}_l$ are disjoint.

We now simplify $\hat{f}^y_{1,i,j}, \hat{f}^z_{1,i,k}, \hat{f}^w_{1,i,l}$ into $f^y_{1,i,j}, f^z_{1,i,k}, f^w_{1,i,l}$ within the same homology, i.e. $g_1 \in \hat{g}_1 + B^1$ for g_1 being $f^y_{1,i,j}$, $f^z_{1,i,k}$, or $f^w_{1,i,l}$. Additionally, we have bound on weights $|g_1| \le |\hat{g}_1|$.

- For $\hat{f}^y_{1,i,j}$, we simplify it into $f^y_{1,i,j}$ which connects the same two qubit through the route next to the seam.

- For $\hat{f}^z_{1,i,k}$, we simplify it into $f^z_{1,i,k}$ which is the shortest path between the qubit and the boundary. This path is next to the seam.

- For $\hat{f}^w_{1,i,l}$, we simplify it into $f^w_{1,i,l}$ which is the shortest path between the qubit and the violated check.

Let $\hat{f}'_{1,i} = \hat{f}^x_{1,i} + \sum_j f^y_{1,i,j} + \sum_k f^z_{1,i,k}$. By construction $\hat{f}'_{1,i}$ is supported next to the seam.

We claim that we can reduce the question of finding $f_{1,i}$ to the question of finding $f'_{1,i}$ for $\hat{f}'_{1,i}$ which will satisfy the inequalities

$$(1)\, |f'_{1,i}|_{\text{int}} \le |\hat{f}'_{1,i}|_{\text{int}}, \quad (2)\, \frac{2}{L}|f'_{1,i}|_{\text{int}} \le |\delta_1 \hat{f}'_{1,i}|_{\text{int}}, \quad (3)\, \frac{1}{2}|\delta_1 f'_{1,i}|_\partial \le |\delta_1 \hat{f}'_{1,i}|_{\text{int}}. \tag{B32}$$

The reason is that suppose we have $f'_{1,i} \in \hat{f}'_{1,i} + B^1$, we can then set $f_{1,i} = f'_{1,i} + \sum_l f^w_{1,i,l}$. One can straightforwardly check that $f_{1,i} \in \hat{f}_{1,i} + B^1$.

We now check inequalities (1), (2), and (3) in Equation (B11). Inequality (1) holds because

$$\begin{aligned} |f_{1,i}| &\le |f'_{1,i}| + \sum_l |f^w_{1,i,l}| \\ &\le |\hat{f}'_{1,i}| + \sum_l |f^w_{1,i,l}| \\ &\le |\hat{f}^x_{1,i}| + \sum_j |f^y_{1,i,j}| + \sum_k |f^z_{1,i,k}| + \sum_l |f^w_{1,i,l}| \\ &\le |\hat{f}^x_{1,i}| + \sum_j |\hat{f}^y_{1,i,j}| + \sum_k |\hat{f}^z_{1,i,k}| + \sum_l |\hat{f}^w_{1,i,l}| \\ &= |\hat{f}_{1,i}| \end{aligned}$$

where the last equality holds because the components are disjoint.

Inequality (2) holds for $\beta_1 = \frac{2}{3L}$ because

$$|f_{1,i}| \le |f'_{1,i}| + \sum_l |f^w_{1,i,l}| \le \frac{L}{2}|\delta_1 \hat{f}'_{1,i}|_{\text{int}} + \sum_l L \le \left(\frac{L}{2} + L\right) |\delta_1 \hat{f}_{1,i}|_{\text{int}}.$$

The second inequality uses $|f^w_{1,i,l}| \le L$. The third inequality uses $|\delta_1 \hat{f}'_{1,i}|_{\text{int}} = |\delta_1 \hat{f}_{1,i}|_{\text{int}}$ and $\sum_l 1 = |\delta_1 \hat{f}_{1,i}|_{\text{int}}$.

Inequality (3) holds for $\eta_1 = \frac{1}{2}$ because

$$|\delta_1 f_{1,i}|_\partial = |\delta_1 f'_{1,i}|_\partial \le 2|\delta_1 \hat{f}_{1,i}|_{\text{int}} = 2|\delta_1 \hat{f}_{1,i}|_{\text{int}}.$$

The remaining task is to find $f'_{1,i}$ for $\hat{f}'_{1,i}$ that is supported next to the seam.

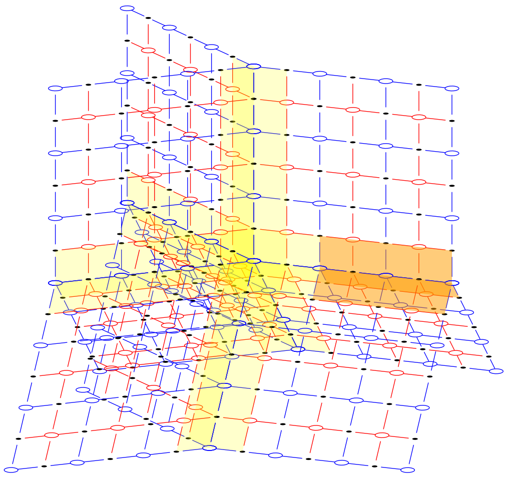

Supplementary Figure 13: The figure illustrate the regions next to the seam in yellow. Each orange region will be referred to as a limb.

e. Step 3: Show the case where $\hat{f}'_{1,i}$ is next to the seam. We simply pick $f'_{1,i}$ to be the vector with the smallest weight in $\hat{f}'_{1,i} + B^1$ while being next to the seam. This automatically satisfies inequality (1) in Equation (B32).

For inequality (2) in Equation (B32), we construct a vector $f''_{1,i} \in \hat{f}'_{1,i} + B^1$ with $\frac{2}{L}|f''_{1,i}|_{\text{int}} \leq |\delta_1 \hat{f}'_{1,i}|_{\text{int}}$. Since $f'_{1,i}$ has the smallest weight, it implies inequality (2). One can simply construct $f''_{1,i}$ by connecting each violated check, i.e. $\text{supp}\delta_1 \hat{f}'_{1,i}$ to the closest boundary. Since each point is at most $\frac{L-1}{2}$ distance away from the boundary, we have $|f''_{1,i}|_{\text{int}} \leq \frac{L-1}{2}|\delta_1 \hat{f}'_{1,i}|_{\text{int}} \leq \frac{L}{2}|\delta_1 \hat{f}'_{1,i}|_{\text{int}}$. (We need the tighter bound $|f''_{1,i}|_{\text{int}} \leq \frac{L-1}{2}|\delta_1 \hat{f}'_{1,i}|_{\text{int}}$ in the next paragraph.)

For inequality (3) in Equation (B32), to show $\frac{1}{2}|\delta_1 f'_{1,i}|_\partial \leq |\delta_1 \hat{f}'_{1,i}|_{\text{int}}$, we use proof by contradiction. We assume $|\delta_1 f'_{1,i}|_\partial > 2|\delta_1 \hat{f}'_{1,i}|_{\text{int}}$ and show that $f'_{1,i}$ is not the vector in $\hat{f}'_{1,i} + B^1$ with the smallest weight. In particular, we will show $|f'_{1,i}| > \frac{L-1}{2}|\delta_1 \hat{f}'_{1,i}|_{\text{int}}$. Because $|f''_{1,i}| \leq \frac{L-1}{2}|\delta_1 \hat{f}'_{1,i}|_{\text{int}}$ from the last paragraph, $f'_{1,i}$ has a larger weight than $f''_{1,i}$ which is the contradiction.

We first perform some basic structural analysis of $f'_{1,i}$ then observe a local condition of $f'_{1,i}$. Consider $v \in \text{supp} f'_{1,i}$ on a horizontal seam. We study the corresponding limb as illustrated in Supplementary Figure 13. The edges emanating from v either end at the neighboring violated check, or go towards the center, or go towards the boundary. Let the number of edges of each type be p, q, r, respectively. We claim that p, q, r satisfies $p + q \geq r$. Otherwise, if $p + q < r$, and one can replace $f'_{1,i}$ with $f'_{1,i} + \delta_0 1_w$, where w is the stabilizer next to the qubit v that is closer to the boundary. See Supplementary Figure 14(a)(b) for an illustration.

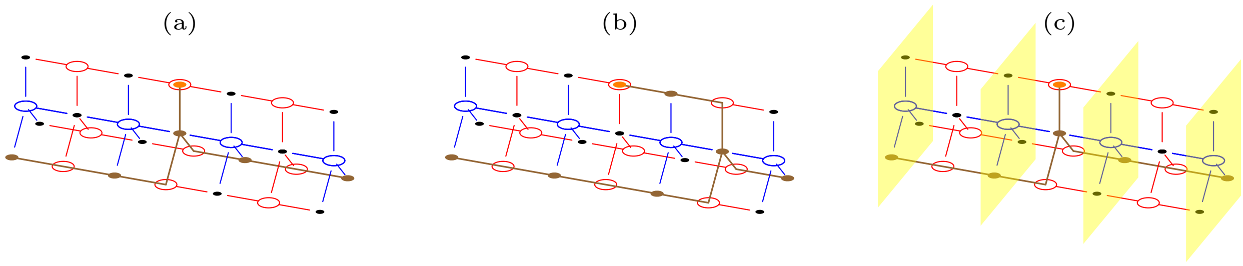

Supplementary Figure 14: The figure illustrates one of the limbs where left is the direction to the center and right is the direction to the boundary. (a) The minimal vector $f'_{1,i}$. (b) The new vector $f'_{1,i} + \delta_0 1_w$ which has a lower weight if $p + q < r$. (c) The cross sections.

We now use the local condition $p + q \geq r$ and the assumption $|\delta_1 f'_{1,i}|_\partial > 2|\delta_1 \hat{f}'_{1,i}|_{\text{int}}$ to show $|f'_{1,i}| >$

$\frac{L-1}{2}|\delta_1 \hat{f}'_{1,i}|_{\text{int}}$. Consider one of the limbs. Suppose the limb contains s violated checks and t lines that connect to the boundary. Consider the number of active qubits on each cross section as shown in Supplementary Figure 14(c). We claim that this number is at least $t-s$ for each cross section. Assuming so, since there are $\frac{L-1}{2}$ cross sections, this limb contains at least $\frac{L-1}{2}(t-s)$ active qubits, i.e. $|\text{supp} f'_{1,i} \cap M| \geq \frac{L-1}{2}(t-s)$ where M is the set of vertices on the limb. We apply this observation to all limbs. Because the total number of violated checks is at most $|\delta_1 \hat{f}'_{1,i}|_{\text{int}}$ and the number of lines that connect to the boundary is $|\delta_1 f'_{1,i}|_\partial$, this means the number of active qubits $|f'_{1,i}|$ is at least $\frac{L-1}{2}(|\delta_1 f'_{1,i}|_\partial - |\delta_1 \hat{f}'_{1,i}|_{\text{int}}) > \frac{L-1}{2}|\delta_1 \hat{f}'_{1,i}|_{\text{int}}$. It suffices to show the claim.

Notice the number of active qubits on the cross section changes only when there is an active qubit on the seam between the two cross sections. With the same notation p, q, r, this number changes from r to q as we go closer to the center. The number drops by at most p because the local condition says $q \geq r - p$. Since the number of active qubits is t on the cross section closest to boundary and the number drops by at most s, each cross section has at least $t-s$ active qubits.
